# Cell Shape and Antibiotic Resistance Are Maintained by the Activity of Multiple FtsW and RodA Enzymes in Listeria monocytogenes

**DOI:** 10.1128/mBio.01448-19

**Published:** 2019-08-06

**Authors:** Jeanine Rismondo, Sven Halbedel, Angelika Gründling

**Affiliations:** a Section of Microbiology, Medical Research Council Centre for Molecular Bacteriology and Infection, Imperial College London, London, United Kingdom; b FG11 Division of Enteropathogenic Bacteria and Legionella, Robert Koch Institute, Wernigerode, Germany; University of Texas Health Science Center at Houston

**Keywords:** FtsW, *Listeria monocytogenes*, RodA, SEDS, antibiotic resistance, cell division

## Abstract

Rod-shaped bacteria have two modes of peptidoglycan synthesis: lateral synthesis and synthesis at the cell division site. These two processes are controlled by two macromolecular protein complexes, the elongasome and divisome. Recently, it has been shown that the Bacillus subtilis RodA protein, which forms part of the elongasome, has peptidoglycan glycosyltransferase activity. The cell division-specific RodA homolog FtsW fulfils a similar role at the divisome. The human pathogen Listeria monocytogenes carries genes that encode up to six FtsW/RodA homologs; however, their functions have not yet been investigated. Analysis of deletion and depletion strains led to the identification of the essential cell division-specific FtsW protein, FtsW1. Interestingly, L. monocytogenes carries a gene that encodes a second FtsW protein, FtsW2, which can compensate for the lack of FtsW1, when expressed from an inducible promoter. L. monocytogenes also possesses three RodA homologs, RodA1, RodA2, and RodA3, and their combined absence is lethal. Cells of a *rodA1 rodA3* double mutant are shorter and have increased antibiotic and lysozyme sensitivity, probably due to a weakened cell wall. Results from promoter activity assays revealed that expression of *rodA3* and *ftsW2* is induced in the presence of antibiotics targeting penicillin binding proteins. Consistent with this, a *rodA3* mutant was more susceptible to the β-lactam antibiotic cefuroxime. Interestingly, overexpression of RodA3 also led to increased cefuroxime sensitivity. Our study highlights that L. monocytogenes genes encode a multitude of functional FtsW and RodA enzymes to produce its rigid cell wall and that their expression needs to be tightly regulated to maintain growth, cell division, and antibiotic resistance.

## Abstract (precis)

The human pathogen Listeria monocytogenes is usually treated with high doses of β-lactam antibiotics, often combined with gentamicin. However, these antibiotics only act bacteriostatically on L. monocytogenes, and the immune system is needed to clear the infection. Therefore, individuals with a compromised immune system are at risk to develop a severe form of *Listeria* infection, which can be fatal in up to 30% of cases. The development of new strategies to treat *Listeria* infections is necessary. Here we show that the expression of some of the FtsW and RodA enzymes of L. monocytogenes is induced by the presence of β-lactam antibiotics, and the combined absence of these enzymes makes bacteria more susceptible to this class of antibiotics. The development of antimicrobial agents that inhibit the activity or production of FtsW and RodA enzymes might therefore help to improve the treatment of *Listeria* infections and thereby lead to a reduction in mortality.

## INTRODUCTION

Bacterial cells are surrounded by a mesh of peptidoglycan (PG) that determines their shape and also protects the cells from lysis due to their high internal turgor pressure (1–3). Peptidoglycan is comprised of glycan strands that are cross-linked by short peptides (4). The glycan strands are composed of alternating *N*-acetylglucosamine and *N*-acetylmuramic acid residues that are connected by a β-1,4 glycosidic bond (5). The synthesis of peptidoglycan begins in the cytoplasm with the production of the PG precursor lipid II by the proteins MurABCDEF, MraY and MurG (6–9). Lipid II is then transported across the cytoplasmic membrane by the flippase MurJ and Amj (10–12) and subsequently incorporated in the growing glycan strand by glycosyltransferases. The polymerization and cross-linking of the glycan strands are facilitated by the activity of glycosyltransferases and transpeptidases, respectively. Class A penicillin binding proteins (PBPs) are bifunctional enzymes that possess glycosyltransferase and transpeptidase activity, whereas class B PBPs contain only a transpeptidase domain (13–15). In addition, some species such as Escherichia coli, Staphylococcus aureus, and Streptococcus pneumoniae carry genes that encode monofunctional glycosyltransferases (MGTs) that can also incorporate lipid II into the growing glycan strand (16–20).

Bacillus subtilis contains genes that encode four class A PBPs and no MGT; however, deletion of all class A PBPs manifests in only small PG changes (21). Recently, it has been shown that members of the SEDS (shape, elongation, division, sporulation) family of proteins, namely, RodA and FtsW, also act as glycosyltransferases (22–25). Both RodA and FtsW form complexes with cognate class B PBPs to enable polymerization and cross-linking of glycan strands (23, 25–28). Interestingly, SEDS proteins and the class B PBPs are more conserved among different bacterial species than class A PBPs are (22).

In rod-shaped bacteria, peptidoglycan is synthesized by two multiprotein complexes, the elongasome that is essential for the cell elongation and the divisome that is crucial for the formation of the division septum (29–32). RodA is part of the elongasome and is essential in many bacteria, including B. subtilis and S. pneumoniae (33, 34). Depletion of RodA results in the production of enlarged, spherical cells in B. subtilis (34). In contrast, FtsW is essential for cell division, and cells depleted for FtsW grow as long filaments (35–37). B. subtilis harbors a sporulation-specific member of the SEDS family, SpoVE in addition to RodA and FtsW. SpoVE is dispensable for growth, but it is essential for the synthesis of the spore cortex peptidoglycan (38, 39). Other *Bacillus* species such as B. cereus and B. anthracis possess four or five FtsW/RodA proteins, and strains of different serotypes of the human pathogen Listeria monocytogenes carry genes that encode even up to six FtsW/RodA homologs in their genome. However, their functions have not yet been investigated.

Here, we determined the roles of the different FtsW and RodA homologs for the growth and cell morphology of L. monocytogenes. Our results show that L. monocytogenes carries genes that encode two FtsW enzymes and three RodA enzymes. The absence of either FtsW1 or of all three RodA proteins is lethal under standard laboratory conditions. L. monocytogenes infections are usually treated with high doses of β-lactam antibiotics such as ampicillin, which inhibit the transpeptidase activity of penicillin binding proteins (PBPs) (40). We demonstrate that the expression of two SEDS proteins, FtsW2 and RodA3, is induced in the presence of β-lactam antibiotics likely to compensate for the inhibition of PBPs and that a *rodA3* mutant is more sensitive to the β-lactam antibiotic cefuroxime. Antimicrobial agents inhibiting the activity of proteins of the SEDS family could therefore potentially improve the treatment of *Listeria* infections in the future.

## RESULTS

### L. monocytogenes 10403S carries genes that encode six FtsW/RodA homologs.

**FIG 1:**
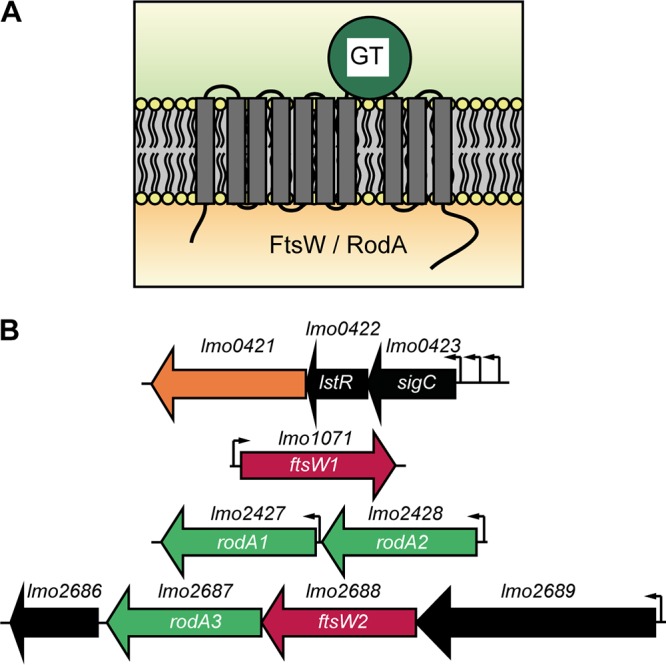
Topology of FtsW and RodA enzymes and their genomic arrangement in L. monocytogenes. (A) Topology models for FtsW and RodA enzymes of L. monocytogenes as predicted using the TMHMM v. 2.0 server (66). GT, glycosyltransferase. (B) Genomic organization of *ftsW* and *rodA* genes in L. monocytogenes. Large arrows indicate the gene orientation. Small black arrows indicate promoters based on the operon structures described by Toledo-Arana et al. (42). Three different promoters have been identified for the *sigC* operon (41).

So far, FtsW and RodA proteins of the human pathogen L. monocytogenes have not been studied. FtsW and RodA are members of the SEDS (shape, elongation, division, sporulation) family of proteins and are multispanning membrane proteins with 8 to 10 transmembrane helices and a large extracellular loop (Fig. 1A). Using BLAST, six proteins with homology to the B. subtilis FtsW and RodA proteins could be identified in the genome of L. monocytogenes 10403S (Table 1). The protein encoded by *lmo0421* has the weakest homology to B. subtilis FtsW and RodA (Table 1; see also Fig. S1 in the supplemental material). *lmo0421* is part of the *sigC* operon, which is comprised of *lmo0422* encoding the PadR-like repressor LstR and *lmo0423* coding for the ECF-type sigma factor SigC (Fig. 1B). The *sigC* operon acts as a lineage II-specific heat shock system (41) and is therefore not present in all L. monocytogenes genomes. Due to the weak homology to FtsW and RodA and its absence in L. monocytogenes strains of lineage I and III, Lmo0421 was excluded from further analysis.

**TABLE 1:** Sequence homology between B. subtilis and L. monocytogenes FtsW/RodA proteins as determined by BLAST^*a*^

L. monocytogenes protein	B. subtilis FtsW (403 aa)^*b*^	B. subtilis RodA (393 aa)^*b*^
Lmo0421 (416 aa)	46/128 aa (36%) [7e−14]	77/350 aa (22%) [4e−09]
FtsW1 (Lmo1071) (402 aa)	195/403 aa (48%) [1e−122]	100/333 aa (30%) [7e−35]
RodA1 (Lmo2427) (391 aa)	124/403 aa (31%) [9e−29]	132/360 aa (37%) [1e−57]
RodA2 (Lmo2428) (389 aa)	112/374 aa (30%) [1e−33]	159/402 aa (40%) [1e−66]
RodA3 (Lmo2687) (369 aa)	95/324 aa (29%) [3e−25]	110/356 aa (31%) [3e−44]
FtsW2 (Lmo2688) (376 aa)	147/342 aa (43%) [5e−67]	95/300 aa (32%) [6e−22]

a The L. monocytogenes proteins and their amino acid sizes (aa) are shown in the leftmost column, and the B. subtilis FtsW (403 aa) and RodA (393 aa) proteins used for the homology search are indicated at the top of the middle and right columns.

b The number of amino acids, which were found to be identical between the respective L. monocytogenes and B. subtilis FtsW/RodA protein are denoted (before the slash) along with the number of amino acids that was utilized by the BLAST algorithm for this comparison (after the slash). The percent identity is given in parentheses, and the corresponding E values are indicated in brackets.

10.1128/mBio.01448-19.1 FIG S1 Sequence alignment of FtsW and RodA proteins from B. subtilis and L. monocytogenes. The alignment was created using Clustal Omega (67). Based on a study by Meeske et al., amino acid residues of B. subtilis RodA that are essential for RodA function are colored in blue, conservative replacements that are possible are shown in yellow, and amino acids that can be changed to certain other amino acids are colored in orange (22). Download FIG S1, EPS file, 1.2 MB. Copyright © 2019 Rismondo et al. 2019 Rismondo et al. This content is distributed under the terms of the Creative Commons Attribution 4.0 International license.

The L. monocytogenes protein Lmo1071 is the closest homolog to B. subtilis FtsW with a sequence identity of 48% (Table 1). Furthermore, L. monocytogenes *lmo1071* and B. subtilis *ftsW* are found in the same chromosomal context. More specifically, *lmo1071* is located between the *lmo1070* gene, which encodes a protein with homology to the B. subtilis YlaN protein, and the *pycA* gene coding for the pyruvate carboxylase, which are also adjacent to *ftsW* in B. subtilis. This analysis suggests that the *lmo1071* gene encodes the cell division protein FtsW. However, L. monocytogenes carries a gene that encodes a second protein, Lmo2688, which shares a higher degree of homology to the B. subtilis FtsW than to the B. subtilis RodA protein (Table 1). Due to these similarities and additional data presented in this study, we refer to Lmo1071 and Lmo2688 as FtsW1 and FtsW2, respectively.

The BLAST search with the B. subtilis RodA sequence as a query sequence yielded the L. monocytogenes protein Lmo2428 as the closest homolog with a sequence identity of 40% (Table 1). In addition to Lmo2428, two additional RodA homologs are present in L. monocytogenes, namely, Lmo2427 and Lmo2687. As presented below, Lmo2427, Lmo2428, and Lmo2687 are likely bona fide RodA homologs and were therefore renamed RodA1, RodA2, and RodA3, respectively. *rodA1* is located adjacent to *rodA2*, but despite their proximity, *rodA1* and *rodA2* are likely not transcribed as part of the same operon (42). In contrast, *rodA3* and *ftsW2* are part of the four-gene operon *lmo2689-lmo2686*. Lmo2689 is similar to a Mg^2+^-type ATPase, whereas *lmo2686* encodes a protein of unknown function. Analysis of around 2,000 genomes of L. monocytogenes strains presently available at NCBI revealed that the five FtsW/RodA homologs named here, FtsW1, FtsW2, RodA1, RodA2, and RodA3, are conserved in the different strains.

### *lmo1071* encodes FtsW1 and is essential for the survival of L. monocytogenes.

**FIG 2:**
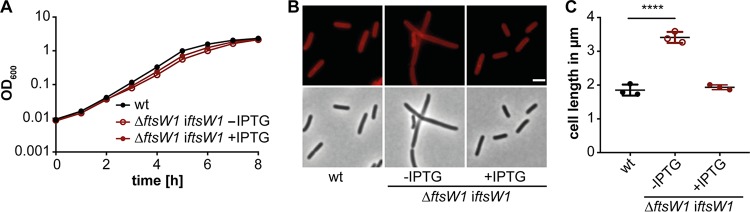
Depletion of FtsW1 results in cell elongation. (A) Growth of the L. monocytogenes FtsW1 depletion strain. Strain 10403SΔ*ftsW1* i*ftsW1* was grown overnight in BHI medium in the presence of 1 mM IPTG. The following day, the cells were washed and used to inoculate fresh BHI medium without IPTG and incubated at 37°C for 8 to 10 h, diluted again, and grown until the next morning. The depletion culture was used to inoculate BHI medium without IPTG or with 1 mM IPTG, and the growth was monitored by determining OD_600_ readings at hourly intervals. Wild-type L. monocytogenes strain 10403S (wt) was used as a control. Averages and standard deviations from three independent experiments were plotted. Of note, due to the small standard deviations, the error bars are not visible in the graph. (B) Microscopy analysis of 10403S (wt) and the *ftsW1* depletion strain grown in the presence or absence of IPTG. For depletion of FtsW1, strain 10403SΔ*ftsW1* i*ftsW1* (the expression of *ftsW1* is controlled by an IPTG-inducible promoter in this strain) was grown as described above for panel A. Cultures of 10403S and 10403SΔ*ftsW1* i*ftsW1* were diluted 1:100 in fresh BHI medium (with 1 mM IPTG where indicated) and grown for 3 h at 37°C, and the cells were subsequently stained with the membrane dye Nile red and analyzed by fluorescence and phase-contrast microscopy. Bar, 2 μm. (C) Cell length measurement of 10403S (wt) and the *ftsW1* depletion strain. The cell length of 300 cells per strain was measured, and the median cell length was calculated and plotted. Three independent experiments were performed, and the average ± standard deviation (error bars) of the median cell length was plotted. For statistical analysis, a one-way analysis of variance (ANOVA) coupled with a Dunnett’s multiple-comparison test was performed. Values that are significantly different (*P* ≤ 0.0001) are indicated by a bar and four asterisks.

The cell division protein FtsW is essential for growth in the Gram-negative and Gram-positive model organisms E. coli and B. subtilis (35, 36, 39, 43). Depletion of FtsW in these organisms leads to a block in cell division and formation of elongated cells (36, 37). All our attempts to delete the *ftsW1* gene in L. monocytogenes 10403S were unsuccessful, suggesting that FtsW1 is also essential for growth in *Listeria*. Next, strain 10403SΔ*ftsW1* i*ftsW1* was constructed, in which the expression of *ftsW1* is controlled by an IPTG-inducible promoter. While no difference in growth was observed between the wild-type and FtsW1 depletion strain (likely due to leakiness of the inducible promoter) (Fig. 2A), cells depleted of FtsW1 were significantly elongated (Fig. 2B and C). Bacteria depleted of FtsW1 had a median cell length of 3.41 ± 0.16 μm, while wild-type and 10403SΔ*ftsW1* i*ftsW1* bacteria grown in the presence of 1 mM IPTG had a median cell length of 1.85 ± 0.16 μm and 1.93 ± 0.07 μm, respectively (Fig. 2C). These data indicate that *lmo1071* encodes the cell division-specific SEDS protein FtsW.

### L. monocytogenes carries a gene that encodes a second FtsW protein.

**FIG 3:**
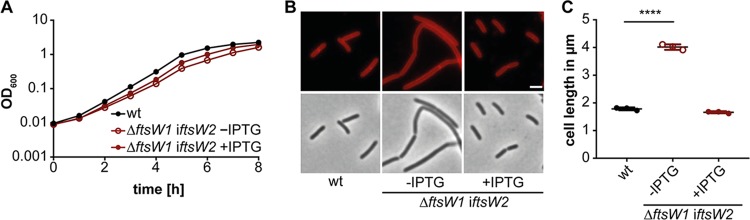
Overexpression of FtsW2 can compensate for the loss of FtsW1. (A) Growth comparison of strains 10403S (wt) and 10403SΔ*ftsW1* i*ftsW2*. Growth curves were performed as described in the legend to Fig. 2. (B) Microscopy images of strains 10403S (wt) and 10403SΔ*ftsW1* i*ftsW2*. For depletion of FtsW2, strain 10403SΔ*ftsW1* i*ftsW2* was grown as described in the legend to Fig. 2. The next morning, cells were diluted 1:100 and grown in BHI broth (with or without IPTG as indicated) until mid-logarithmic growth phase, stained with the membrane dye Nile red, and subjected to fluorescence and phase-contrast microscopy analysis. Bar, 2 μm. (C) Cell length measurements of 10403S (wt) and 10403SΔ*ftsW1* i*ftsW2*. The cell length of 300 cells per strain was measured, and the median cell length was calculated and plotted. Three independent experiments were performed, and the average and standard deviation of the median cell length were plotted. For statistical analysis, a one-way ANOVA coupled with a Dunnett’s multiple-comparison test was performed (****, *P* ≤ 0.0001).

To our knowledge, all bacteria analyzed so far possess only one FtsW protein that is essential for cell survival. We identified a second potential FtsW protein, Lmo2688, in L. monocytogenes. In contrast to *ftsW1*, an L. monocytogenes *ftsW2* deletion strain could be constructed, and no significant growth or cell morphology phenotypes could be observed for the Δ*ftsW2* deletion strain (Fig. S2). In a previous study, it was reported that the operon comprised of genes *lmo2689-lmo2686* is only minimally expressed when L. monocytogenes 10403S is grown in BHI broth (44). We reasoned that if *ftsW2* does indeed code for a second FtsW protein, it should be possible to delete *ftsW1* in a strain in which *ftsW2* is artificially expressed from an IPTG-inducible promoter. Indeed, strain 10403SΔ*ftsW1* i*ftsW2* could be generated in the presence of IPTG. In contrast, we were unable to generate strain 10403SΔ*ftsW1* when any of the other FtsW/RodA homologs Lmo2427 (RodA1), Lmo2428 (RodA2), or Lmo2687 (RodA3) were expressed from the same IPTG-inducible promoter system. While prolonged depletion of FtsW2 in strain 10403SΔ*ftsW1* i*ftsW2* again had no impact on growth, the cells were significantly elongated in the absence of the inducer compared to the wild type or bacteria grown in the presence of inducer (Fig. 3). These data strongly suggest that *ftsW2* encodes a second FtsW enzyme, while the remaining three proteins, Lmo2427, Lmo2428, and Lmo2687, likely function as RodA proteins.

10.1128/mBio.01448-19.2 FIG S2 An L. monocytogenes *ftsW2* mutant does not show a growth or cell length defect. (A) Growth of L. monocytogenes strains 10403S (wt) and 10403SΔ*ftsW2* in BHI broth at 37°C. (B) Microscopy images of wild-type and *ftsW2* mutant L. monocytogenes strains. Bacteria from cultures of the strains described in panel A were stained with the membrane dye Nile red and analyzed by fluorescence and phase-contrast microscopy. Bar, 2 μm. (C) Cell length measurement. The cell length of 300 cells of strain 10403S (wt) and 10403SΔ*ftsW2* was measured, and the median cell length was calculated and plotted. Three independent experiments were performed, and the average and standard deviation of the median cell length were plotted. Download FIG S2, TIF file, 3.0 MB. Copyright © 2019 Rismondo et al. 2019 Rismondo et al. This content is distributed under the terms of the Creative Commons Attribution 4.0 International license.

### L. monocytogenes carries genes that encode three RodA homologs.

**FIG 4:**
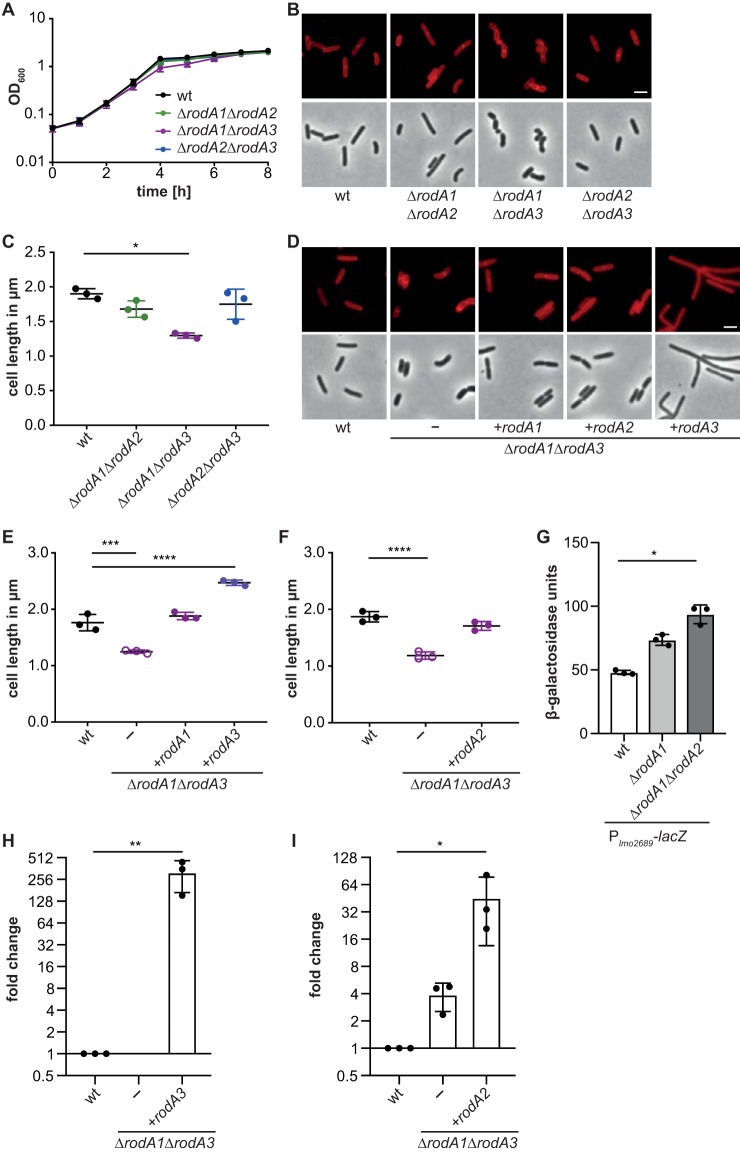
RodA1 and RodA3 are essential to maintain the rod shape of L. monocytogenes. (A) Growth of L. monocytogenes 10403S (wt), 10403SΔ*rodA1*Δ*rodA2*, 10403SΔ*rodA1*Δ*rodA3*, and 10403SΔ*rodA2*Δ*rodA3* strains in BHI broth at 37°C. (B) Microscopy images of wt and mutant L. monocytogenes strains. Cells from the strains described above for panel A were stained with the membrane dye Nile red and analyzed by fluorescence and phase-contrast microscopy. Bar, 2 μm. (C) Cell length measurements of wt and mutant L. monocytogenes strains. The cell length of 300 cells per strain was measured, and the median cell length was calculated and plotted. Three independent experiments were performed, and the average and standard deviation of the median cell length was plotted. For statistical analysis, a one-way ANOVA coupled with a Dunnett's multiple comparison test was used (*, *P* ≤ 0.05). (D) Microscopy images of wild-type and mutant L. monocytogenes strains. Cells from 10403S (wt), 10403SΔ*rodA1*Δ*rodA3* (−), and 10403SΔ*rodA1*Δ*rodA3* expressing *rodA1* (+*rodA1*), *rodA2*, or *rodA3* from an IPTG-inducible promoter were stained with the membrane dye Nile red, and analyzed by fluorescence and phase-contrast microscopy. Bar, 2 μm. (E and F) Cell length analysis of 10403SΔ*rodA1*Δ*rodA3* complemented strains. Analysis was performed as described above for panel C. (G) β-Galactosidase assay using L. monocytogenes strains 10403S (wt), 10403SΔ*rodA1*, and 10403SΔ*rodA1*Δ*rodA2* carrying a P*_lmo2689_*-*lacZ* fusion construct. Averages and standard deviations of three independent experiments were plotted. For statistical analysis, a one-way ANOVA coupled with a Dunnett’s multiple-comparison test was used (*, *P* ≤ 0.05). (H) Analysis of *rodA3* expression by qRT-PCR. RNA was isolated from strains 10403S (wt), 10403SΔ*rodA1*Δ*rodA3* (−), and 10403SΔ*rodA1*Δ*rodA3* i*rodA3* grown in the presence of IPTG. Expression of *rodA3* was normalized to the expression of *gyrB*, and fold changes were calculated using the ΔΔ*C_T_* method. Averages and standard deviations of three independent experiments were plotted. For statistical analysis, a one-way ANOVA coupled with a Dunnett’s multiple-comparison test was performed (**, *P* ≤ 0.01). (I) Analysis of *rodA2* expression using qRT-PCR. Same as panel H but using strains 10403S, 10403SΔ*rodA1*Δ*rodA3* (−), and 10403SΔ*rodA1*Δ*rodA3* i*rodA2* grown in the presence of IPTG. Averages and standard deviations of three independent experiments were plotted. For statistical analysis, a one-way ANOVA coupled with a Dunnett’s multiple-comparison test was performed (*, *P* ≤ 0.05).

We were able to assign roles for two of the FtsW/RodA homologs as FtsW-like proteins. However, L. monocytogenes carries genes that encode three additional homologs, which show a higher similarity to the B. subtilis RodA protein compared to the B. subtilis FtsW protein (Table 1). As described above, expression of none of the enzymes Lmo2427, Lmo2428, or Lmo2687 was able to rescue the growth of an *ftsW1* deletion strain, indicating that these enzymes likely function as RodA proteins in L. monocytogenes, and hence, they were renamed RodA1, RodA2, and RodA3, respectively. All attempts to construct a *rodA1 rodA2 rodA3* triple mutant failed, further corroborating that these proteins function as RodA proteins and that at least one of them needs to be present for cell viability. To determine whether the different RodA homologs have distinct functions or whether they are merely duplications, single and double mutant strains were generated. No significant differences with regard to growth and cell length could be observed between the wild-type strain 10403S and single *rodA1*, *rodA2*, or *rodA3* deletion strains (Fig. S3). Similar observations were made with the *rodA* double mutant strains 10403SΔ*rodA1*Δ*rodA2* and 10403SΔ*rodA2*Δ*rodA3* (Fig. 4). However, cells lacking RodA1 and RodA3 were shorter (1.3 ± 0.03 μm) than wild-type cells (1.9 ± 0.06 μm; Fig. 4B and C), indicating that either RodA1 or RodA3 needs to be present for L. monocytogenes to maintain its rod shape. RodA3 is part of the *lmo2689-lmo2686* operon that is only minimally expressed when L. monocytogenes is grown in BHI broth (44). The fact that we observe differences in cell morphology between a *rodA1* single mutant and the *rodA1 rodA3* double mutant suggests that the function of the two proteins could be additive or that *rodA3* expression, which is only minimally expressed in a wild-type strain under standard laboratory growth conditions, might increase upon deletion of *rodA1*. To test whether *rodA3* expression is increased in the absence of other RodA proteins, we fused the promoter upstream of *lmo2689* and driving *rodA3* expression to *lacZ* and inserted this fusion into the chromosome of wild-type 10403S, the *rodA1* and *rodA1 rodA2* deletion strains. The promoter activity was indeed 1.5- to 2-fold higher in the *rodA1* and *rodA1 rodA2* mutant strains compared to the wild type, as assessed by the increase in the β-galactosidase activity (Fig. 4G). This result indicates that expression of the *lmo2689-lmo2686* operon, which encodes FtsW2 and RodA3, is induced in the absence of RodA1, suggesting a coordination of the expression of the different RodA homologs.

10.1128/mBio.01448-19.3 FIG S3 Single *rodA1*, *rodA2*, or *rodA3* L. monocytogenes deletion strains do not show a growth or cell length defect. (A) Growth of L. monocytogenes 10403S (wt), 10403SΔ*rodA1*, 10403SΔ*rodA2*, and 10403SΔ*rodA3* in BHI broth at 37°C. (B) Microscopy images of wild-type and mutant L. monocytogenes strains. Bacteria from cultures of the strains described in panel A were stained with the membrane dye Nile red and analyzed by fluorescence and phase-contrast microscopy. Bar, 2 μm. (C) Cell length of L. monocytogenes wt and *rodA* single mutants. The cell length of 300 cells per strain was measured, and the median cell length was calculated and plotted. Three independent experiments were performed, and the average and standard deviation of the median cell length were plotted. Download FIG S3, TIF file, 4.2 MB. Copyright © 2019 Rismondo et al. 2019 Rismondo et al. This content is distributed under the terms of the Creative Commons Attribution 4.0 International license.

To confirm that the decrease in cell length of the double mutant strain 10403SΔ*rodA1*Δ*rodA3* depends on the absence of RodA1 and RodA3, complemented strains with IPTG-inducible expression of *rodA1* or *rodA3* were constructed. Expression of RodA1 restored the cell length to 1.84 ± 0.1 μm, which is comparable to the cell length of wild-type cells (1.76 ± 0.15 μm; Fig. 4E). On the other hand, expression of *rodA3* from an ectopic locus and IPTG-inducible promoter in strain 10403SΔ*rodA1*Δ*rodA3* led to the formation of longer cells with an average cell length of 2.47 ± 0.01 μm (Fig. 4D and E). These results indicate that induction of RodA3 from the IPTG-inducible promoter likely results in overproduction of the protein compared to expression from the native promoter. To confirm that *rodA3* expression is increased when expressed from the IPTG-inducible promoter compared to its native promoter, *rodA3* transcript levels were assessed by qRT-PCR in the wild-type strain, the 10403SΔ*rodA1*Δ*rodA3* deletion strain, and strain 10403SΔ*rodA1*Δ*rodA3* i*rodA3* grown in the presence of IPTG (Fig. 4H). Significant higher *rodA3* transcript levels were detected in the inducible strain in the presence of IPTG compared to the wild-type strain. Similarly, expression of *rodA3* from an IPTG-inducible promoter in wild-type 10403S led to the formation of elongated cells with an average cell length of 2.49 ± 0.08 μm, whereas additional expression of *rodA1* or *rodA2* using the same inducible system had no impact on the cell length of 10403S (Fig. S4). These results highlight that in particular fine-tuning of RodA3 production is essential for cell length determination in L. monocytogenes.

10.1128/mBio.01448-19.4 FIG S4 Overexpression of RodA3 results in cell elongation. (A) Cell length measurements of 10403S (wt) and strains 10403S pIMK3-*rodA1*, 10403S pIMK3*-rodA2*, and 10403S pIMK3-*rodA3.* The cell length of 300 cells per strain was measured. Three independent experiments were performed, and the average and standard deviation of the median cell length were plotted. For statistical analysis, a one-way ANOVA coupled with a Dunnett’s multiple-comparison test was performed (****, *P* ≤ 0.0001). (B) Microscopy images of L. monocytogenes strains. Bacteria from cultures of the strains described in panel A were stained with the membrane dye Nile red and analyzed by fluorescence and phase-contrast microscopy. Bar, 2 μm. Download FIG S4, TIF file, 2.7 MB. Copyright © 2019 Rismondo et al. 2019 Rismondo et al. This content is distributed under the terms of the Creative Commons Attribution 4.0 International license.

The observation that the *rodA1 rodA3* double mutant forms shorter cells suggests that RodA2 is not sufficient to maintain the cell length of L. monocytogenes. There are several possible explanations for this. RodA2 might have a reduced activity compared to RodA1 or RodA3. RodA2 might have a function that is different from the functions of RodA1 and RodA3 or the protein levels of RodA2 might be sufficient to maintain cell viability but too low to maintain the rod shape. To investigate this further, a strain was constructed that lacks *rodA1* and *rodA3* but carries pIMK3-*rodA2* to allow for IPTG-inducible expression of *rodA2* in addition to the expression of *rodA2* from its native locus (10403SΔ*rodA1*Δ*rodA3* i*rodA2*). In the absence of the inducer, the cells had a median cell length of 1.2 ± 0.03 μm (data not shown). However, the cell length of strain 10403SΔ*rodA1*Δ*rodA3* i*rodA2* increased to 1.71 ± 0.8 μm when the strain was grown in the presence of IPTG (Fig. 4F). Therefore, additional expression of *rodA2*, which was verified by qRT-PCR (Fig. 4I), can partially complement the cell length phenotype of the *rodA1 rodA3* deletion strain, suggesting that RodA2 has a function similar to that of RodA1 and RodA3 but that it has either a lower activity or is not expressed in sufficient amounts from its native promoter for proper cell length maintenance.

**FIG 5:**
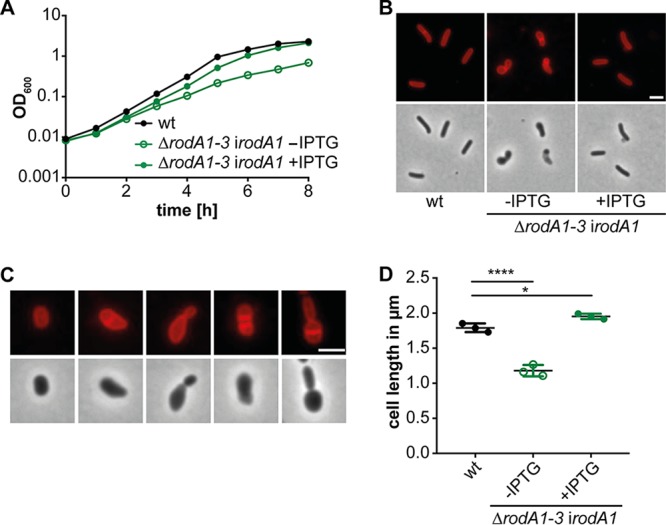
Absence of all three RodA proteins leads to a drastic cell shortening. (A) Growth of L. monocytogenes in the absence of all three RodA proteins. Growth curves were performed as described in the legend to Fig. 2 using strain 10403S and strain 10403SΔ*rodA1-3* i*rodA1* grown in the presence (+) or absence (−) of IPTG. (B and C) Microscopy analysis of strain 10403SΔ*rodA1-3* i*rodA1*. For depletion of RodA1, strain 10403SΔ*rodA1-3* i*rodA1* was grown in the same way as described for the FtsW1 depletion in the legend to Fig. 2. (B) Wild-type 10403S and depleted 10403SΔ*rodA1-3* i*rodA1* cultures were diluted 1:100, grown for 3 h at 37°C (where indicated in the presence of 1 mM IPTG) and stained with Nile red, and phase-contrast and fluorescence microscopy images were taken. Bar, 2 μm. (C) Microscopy images showing examples of different cell morphologies observed for strain 10403SΔ*rodA1-3* i*rodA1* depleted for RodA1. Bar, 2 μm. (D) Cell length measurements of 10403S (wt) and strain 10403SΔ*rodA1-3* i*rodA1.* The cell length of 300 cells per strain was measured. Three independent experiments were performed, and the average and standard deviation of the median cell length was plotted. For statistical analysis, a one-way ANOVA coupled with a Dunnett’s multiple-comparison test was performed (*, *P* ≤ 0.05; ****, *P* ≤ 0.0001).

As stated above, several attempts to construct a strain inactivated for all three RodA homologs remained unsuccessful, suggesting that at least one of the proteins RodA1, RodA2, or RodA3 needs to be present for the viability of L. monocytogenes. The results presented so far indicate that RodA1 is the most important RodA homolog considering that RodA2 alone is not sufficient to maintain the rod shape and that RodA3 is only minimally expressed under standard laboratory conditions (44). To understand the impact of RodA enzymes on cell growth and cell division in L. monocytogenes, a strain was constructed that lacks all three *rodA* genes from its genome but harbors pIMK3-*rodA1* to enable IPTG-inducible expression of RodA1 (strain 10403SΔ*rodA1-3* i*rodA1*). Prolonged depletion of RodA1 in strain 10403SΔ*rodA1-3* i*rodA1* led to a growth defect that was not seen when the strain was grown in the presence of the inducer (Fig. 5A). However, the depletion was not efficient enough to see a complete growth inhibition, which would be expected for a strain lacking all three RodA homologs. This is likely caused by residual *rodA1* expression from the inducible promoter even in the absence of IPTG. Consistent with this notion, even after prolonged depletion, *rodA1* transcripts could still be detected in strain 10403SΔ*rodA1-3* i*rodA1* as assessed by qRT-PCR (Fig. S5). However, cells of the L. monocytogenes strain 10403SΔ*rodA1-3* i*rodA1* that were grown without IPTG were significantly shorter with a cell length of 1.18 ± 0.08 μm compared to cells of the double mutant 10403SΔ*rodA1*Δ*rodA3* or the wild-type strain 10403S (Fig. 5B and D). Interestingly, different cell morphologies could be observed for strain 10403SΔ*rodA1-3* i*rodA1* after prolonged RodA1 depletion (Fig. 5C). The placement of the division septum was affected in some cells and daughter cells of different sizes, or cells with two septa were observed (Fig. 5C). These morphological defects could be complemented, and the cell length increased to 1.95 ± 0.04 μm upon the addition of IPTG and expression of RodA1 (Fig. 5D). These data highlight that RodA1 alone is sufficient to maintain the cell shape of L. monocytogenes.

10.1128/mBio.01448-19.5 FIG S5 *rodA1* transcripts are still detected even after prolonged RodA1 depletion. Analysis of *rodA1* expression using qRT-PCR. RNA was isolated from strains 10403S and 10403SΔ*rodA1*-*3* i*rodA1* that had been grown in the presence and absence of IPTG. Expression of *rodA1* was normalized to the expression of *gyrB*, and fold changes were calculated using the ΔΔCt method. Averages and standard deviations of three independent experiments were plotted. For statistical analysis, a one-way ANOVA coupled with a Dunnett’s multiple-comparison test was performed (***, *P* ≤ 0.001). Download FIG S5, TIF file, 1.6 MB. Copyright © 2019 Rismondo et al. 2019 Rismondo et al. This content is distributed under the terms of the Creative Commons Attribution 4.0 International license.

### Decreased moenomycin and lysozyme resistance in the absence of RodA homologs.

Next, we wondered whether the absence of FtsW or RodA proteins affects the resistance of L. monocytogenes toward the antibiotics penicillin, bacitracin, and moenomycin, which target different steps in the peptidoglycan biosynthesis process. Penicillin binds to the transpeptidase domain of PBPs and inhibits their function, leading to a reduced cross-linking of the peptidoglycan (45, 46). Bacitracin inhibits the dephosphorylation of the bactoprenol carrier, leading to a block in lipid II synthesis (47). The phosphoglycolipid antibiotic moenomycin inhibits the glycosyltransferase activity of bifunctional PBPs and thereby prevents the polymerization of the glycan chain (48).

**FIG 6:**
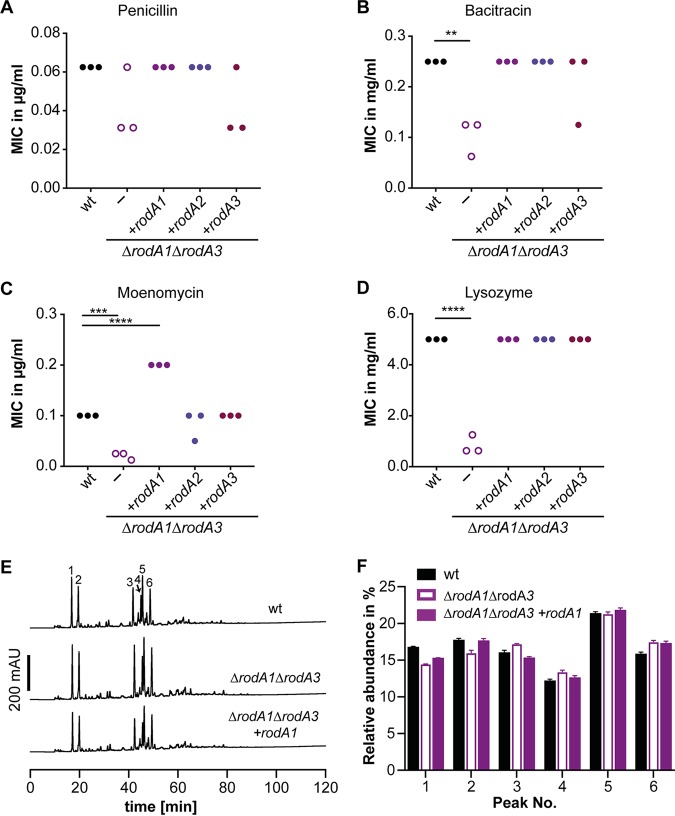
Impact of *rodA1* and *rodA3* deletion on the antibiotic and lysozyme resistance. (A to D) The MICs for the antibiotics penicillin G (A), bacitracin A (B), moenomycin (C), and lysozyme (D) were determined for the wild-type L. monocytogenes strain 10403S, the 10403SΔ*rodA1*Δ*rodA3* deletion strain, and the complemented strains 10403SΔ*rodA1*Δ*rodA3* i*rodA1*, 10403SΔ*rodA1*Δ*rodA3* i*rodA2*, and 10403SΔ*rodA1*Δ*rodA3* i*rodA3* using a broth microdilution assay. The complemented strains were grown in the presence of 1 mM IPTG. The result of three biological replicates are shown in panels A to D. For statistical analysis, a one-way ANOVA coupled with a Dunnett’s multiple-comparison test was used (**, *P* ≤ 0.01; ***, *P* ≤ 0.001; ****, *P* ≤ 0.0001). (E) HPLC analysis of the muropeptide composition of 10403S (wt), 10403SΔ*rodA1*Δ*rodA3*, and 10403SΔ*rodA1*Δ*rodA3* i*rodA1* (grown in the presence of IPTG to induce expression of RodA1). The major muropeptide peaks are numbered 1 to 6 as previously described (51, 52). (F) Relative abundance of muropeptide peaks 1 to 6 in peptidoglycan isolated from strains 10403S (wt), 10403SΔ*rodA1*Δ*rodA3*, and 10403SΔ*rodA1*Δ*rodA3* i*rodA1*. Average values and standard deviations were calculated from three independent peptidoglycan extractions.

No significant differences could be observed in terms of resistance against penicillin, bacitracin, or moenomycin for the FtsW1 depletion strain 10403SΔ*ftsW1* i*ftsW1*. This is presumably due to basal level expression of *ftsW1* even in the absence of the inducer. Simultaneous deletion of *rodA1* and *rodA3* resulted in a slight decrease in the MIC for penicillin; however, this difference was not significant (Fig. 6A). On the other hand, strain 10403SΔ*rodA1*Δ*rodA3* was two- to fourfold more sensitive to the antibiotic bacitracin (Fig. 6B). This phenotype could be complemented by expressing either RodA1, RodA2, or RodA3 from an IPTG-inducible promoter (Fig. 6B).

As described above, moenomycin inhibits the transglycosylase activity of PBPs, leading to a decreased activity of these enzymes. In the absence of RodA1 and RodA3, cells are more susceptible to reduced activity of PBPs, manifesting in a fourfold-reduced resistance to moenomycin (Fig. 6C). Induction of RodA1 expression in strain 10403SΔ*rodA1*Δ*rodA3* resulted in a significantly higher resistance to moenomycin compared to that of wild-type strain 10403S, and expression of RodA2 or RodA3 led to partial or complete complementation of the moenomycin sensitivity (Fig. 6C).

Moreover, resistance to lysozyme, an enzyme that cleaves the linkage between *N*-acetylmuramic acid and *N*-acetylglucosamine residues of the peptidoglycan, was drastically decreased in strain 10403SΔ*rodA1*Δ*rodA3* and could be fully restored by expression of RodA1, RodA2, or RodA3 (Fig. 6D). Lysozyme resistance in L. monocytogenes is mainly accomplished by two modifications of the peptidoglycan; deacetylation of *N*-acetylglucosamine residues by PgdA or O-acetylation of *N*-acetylmuramic acid residues by OatA (49, 50). To determine whether the activity of PgdA is changed in the absence of RodA1 and RodA3, peptidoglycan was purified from the 10403SΔ*rodA1*Δ*rodA3* mutant strain and digested with mutanolysin, and the resulting muropeptides were analyzed by HPLC. Peptidoglycan samples isolated from the wild-type strain 10403S and the complementation strain 10403SΔ*rodA1*Δ*rodA3* i*rodA1* that had been grown in the presence of IPTG were analyzed as controls (Fig. 6E). The main muropeptide peaks were assigned as described previously (51, 52). Peaks 1 and 2 correspond to the acetylated and deacetylated monomeric muropeptides, respectively, whereas peak 3 and peaks 4 to 6 are acetylated and deacetylated muropeptide dimers, respectively. Deletion of *rodA1* and *rodA3* led to a reduction of both monomeric muropeptides and therefore to an increase in cross-linked peptidoglycan fragments by approximately 2% compared to the wild-type strain 10403S, in which 65% of the peptidoglycan was cross-linked (Fig. 6F). However, no significant difference with regard to the deacetylated muropeptides could be observed between wild-type 10403S, strain 10403SΔ*rodA1*Δ*rodA3*, and the 10403SΔ*rodA1*Δ*rodA3* i*rodA1* complementation strain (Fig. 6F). These results suggest that the lysozyme sensitivity phenotype of strain 10403SΔ*rodA1*Δ*rodA3* is not caused by changes in the peptidoglycan deacetylation but instead is due to general defects in the peptidoglycan structure.

### Cell wall-acting antibiotics induce the promoter of *lmo2689*.

**FIG 7:**
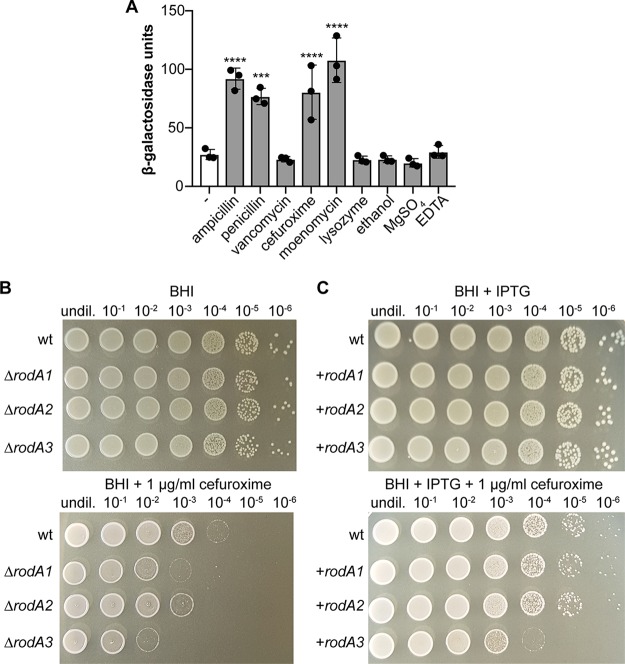
Cell wall-acting antibiotics increase the *lmo2689* promoter activity. (A) Bacteria from mid-logarithmic cultures of strain 10403S pPL3e-P*_lmo2689_*-*lacZ* were exposed for 2 h at 37°C to different stressors. The activity of the *lmo2689* promoter was subsequently determined by performing β-galactosidase activity assays as described in Materials and Methods. Bacteria that had been grown in the absence of a stressor were included as a negative control (−). The averages of the β-galactosidase activity units and standard deviations from three independent experiments were plotted. For statistical analysis, a one-way ANOVA coupled with a Dunnett’s multiple-comparison test was used (***, *P* ≤ 0.001; ****, *P* ≤ 0.0001). (B and C) Absence and overexpression of RodA3 result in decreased cefuroxime resistance. Dilutions of overnight cultures of strains 10403S (wt), 10403SΔ*rodA1*, 10403SΔ*rodA2*, and 10403SΔ*rodA3* (B) and strains 10403S (wt), 10403S pIMK3-*rodA1*, 10403S pIMK3-*rodA2*, and 10403S pIMK3-*rodA3* (C) were spotted on BHI agar and BHI agar containing 1 μg/ml cefuroxime and incubated for 24 h at 37°C. BHI agar plates were supplemented with 1 mM IPTG for cefuroxime resistance assays shown in panel C. A representative result from three independent experiments is shown. undil., undiluted.

The operon *lmo2689-lmo2686*, which contains the genes encoding FtsW2 and RodA3, is only minimally expressed under standard laboratory conditions (44). A genome-wide transcriptional analysis performed in L. monocytogenes strain LO28 has shown that *lmo2687, lmo2688*, and *lmo2689* are part of the CesR regulon (53). The cephalosporin sensitivity response regulator CesR is part of the CesRK two-component system that regulates the transcription of several cell envelope-related genes in response to changes in cell wall integrity, such as those caused by the presence of cell wall-acting antibiotics or alcohols, including ethanol (53–55). Therefore, we next used the *lmo2689* promoter*-lacZ* fusion described above to assess whether expression of the *lmo2689-lmo2686* operon is induced in the presence of antibiotics that target different processes of the PG biosynthesis or ethanol. Indeed, increased β-galactosidase activity could be measured for cells that had been grown in the presence of subinhibitory concentrations of the β-lactam antibiotics ampicillin, penicillin, and cefuroxime and the phosphoglycolipid moenomycin (Fig. 7A). In contrast, no increase in β-galactosidase activity could be detected upon the addition of vancomycin, lysozyme, or ethanol compared to untreated control cells (Fig. 7A). We also tested whether the presence of MgSO_4_ or EDTA has an impact on *lmo2689* promoter activity, since *lmo2689* encodes a putative Mg^2+^-type ATPase. However, the β-galactosidase activity of cells grown in the presence of MgSO_4_ or EDTA was comparable to the β-galactosidase activity seen for untreated cells (Fig. 7A). These results indicate that the expression of *ftsW2* and *rodA3*, which are part of the *lmo2689-lmo2686* operon, are induced in the presence of various cell wall-acting antibiotics, suggesting that FtsW2 and RodA3 might be important for the intrinsic resistance of L. monocytogenes against these antibiotics. However, no significant differences in MICs for penicillin and moenomycin could be observed between wild-type 10403S, the *ftsW2* or *rodA3* single mutant strains, or the *ftsW2 rodA3* double mutant (Fig. S6). However, there was a slight reduction in the resistance of the *rodA3* single mutant against cefuroxime compared to the wild type (Fig. S6). To further assess whether there is a difference in the cefuroxime resistance between the L. monocytogenes wild-type strain 10403S and the *rodA1*, *rodA2*, and *rodA3* single mutant strains, dilutions of overnight cultures were spotted on BHI agar plates with or without 1 μg/ml cefuroxime. Deletion of *rodA1* or *rodA2* results in a slightly reduced ability of these strains to grow on BHI plates supplemented with 1 μg/ml cefuroxime compared to the wild-type 10403S strain (Fig. 7B). However, deletion of *rodA3* leads to a stronger reduction of growth on BHI plates containing 1 μg/ml cefuroxime compared to the growth of *rodA1* and *rodA2* single mutants (Fig. 7B). Interestingly, overexpression of RodA3, but not of RodA1 or RodA2, also resulted in decreased resistance toward cefuroxime compared to the wild-type strain 10403S (Fig. 7C). Our results therefore suggest that L. monocytogenes induces the expression of *rodA3* and *ftsW2* in the presence of β-lactam antibiotics and moenomycin to compensate for the inhibition of the glycosyltransferase and transpeptidase activity of PBPs. In particular, RodA3 seems to play an important function for the intrinsic cephalosporin resistance in L. monocytogenes, and its expression needs to be finely balanced, as both its absence as well as increased expression have detrimental effects on resistance against this antibiotic.

10.1128/mBio.01448-19.6 FIG S6 Deletion of *rodA3* but not *ftsW2* has a minor impact on the resistance toward a selected number of cell wall-active antibiotics. The minimal inhibitory concentrations for the antibiotics penicillin G (A), moenomycin (B), and cefuroxime (C) were determined for the wild-type L. monocytogenes strain 10403S, the 10403SΔ*rodA3* and 10403SΔ*ftsW2* single deletion strains, and the 10403SΔ*ftsW2*Δ*rodA3* double mutant strain using a microbroth dilution assay. The results of three biological replicates are shown in panels A to C. Download FIG S6, TIF file, 4.0 MB. Copyright © 2019 Rismondo et al. 2019 Rismondo et al. This content is distributed under the terms of the Creative Commons Attribution 4.0 International license.

### FtsW and RodA proteins interact with class B PBPs.

**FIG 8:**
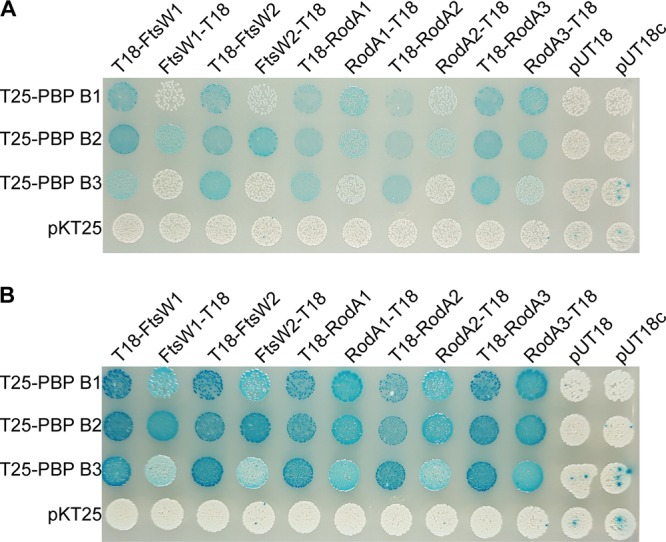
FtsW and RodA enzymes interact with class B PBPs. (A and B) FtsW/RodA and class B PBPs were fused to the T18 and T25 fragments of the Bordetella pertussis adenylate cyclase and cotransformed into the bacterial two-hybrid strain BTH101. Cotransformations with pKT25, pUT18, and pUT18c were used as negative controls. The plates were photographed after incubation at 30°C for 24 h (A) or 48 h (B). Representative images from four independent experiments are shown.

Previous studies have shown that members of the SEDS protein family act together with a cognate class B PBP to synthesize and cross-link peptidoglycan (28, 34, 56–58). L. monocytogenes carries genes that encode three class B PBPs, namely PBP B1, PBP B2, and PBP B3. To identify potential protein-protein interactions between the L. monocytogenes class B PBPs and the FtsW/RodA proteins, a bacterial adenylate cyclase two-hybrid (BACTH) analysis was performed. Interactions were detected between the three L. monocytogenes PBP B1, PBP B2, and PBP B3 and all FtsW and RodA homologs (Fig. 8). These results suggest that the FtsW and RodA proteins also form a complex with class B PBPs in L. monocytogenes. However, using this bacterial two-hybrid approach, it was not possible to determine specific SEDS protein and class B PBP pairs.

## DISCUSSION

Bacterial cell elongation and cell division need to be tightly regulated to maintain cell shape. This is accomplished by two multiprotein complexes, the elongasome and divisome, which are coordinated by the actin homolog MreB and the tubulin homolog FtsZ, respectively (30–32, 59, 60). The SEDS protein FtsW is part of the divisome and essential for growth as shown for many bacteria, including E. coli, B. subtilis, and S. aureus (28, 35, 36, 39, 43). Our experiments suggested that FtsW1 is also essential in L. monocytogenes; however, a second FtsW protein, FtsW2, can compensate for the loss of FtsW1 if it is expressed from an inducible promoter. FtsW2 is encoded by a gene in the *lmo2689-lmo2686* operon that appears to be only minimally expressed when L. monocytogenes 10403S is grown under standard laboratory conditions (44). The expression of the *lmo2689-lmo2686* operon is regulated by the two-component system CesRK that is activated by cell envelope stress (53–55). Using an L. monocytogenes strain carrying a P*_lmo2689_*-*lacZ* promoter fusion, we could detect increased β-galactosidase activity after incubation with subinhibitory concentrations of different β-lactam antibiotics, including penicillin, cefuroxime, and moenomycin. However, expression of the *lmo2689-lmo2686* operon was not induced by other cell wall-targeting antibiotics such as vancomycin or the hydrolase lysozyme. This suggests that inhibition of the glycosyltransferase or transpeptidase activity of PBPs leads to activation of the *lmo2689-lmo2686* operon, and hence, to the expression of *ftsW2* as well as *rodA3*.

The rod shape-determining protein RodA is part of the elongation machinery. The data presented in this study suggest that L. monocytogenes carries genes that encode not one but three RodA proteins and that depletion of the three RodA enzymes leads to a decreased cell length (Fig. 5). Simultaneous deletion of *rodA1* and *rodA3* already results in the formation of shorter cells, whereas cells of strains with *rodA1* and *rodA2* or *rodA2* and *rodA3* deleted have a cell length that is comparable to that of the wild-type strain 10403S. Taking into consideration that *rodA3* is only minimally expressed under standard laboratory growth conditions in L. monocytogenes 10403S (44), the results presented in this study suggest that *rodA3* expression becomes induced upon inactivation of RodA1, since we observed morphological differences between the *rodA1* single mutant and *rodA1 rodA3* double mutant strains. Indeed, β-galactosidase assays confirmed that deletion of *rodA1* or *rodA1 rodA2* increases the activity of the promoter from which *rodA3* is expressed. The data presented in this study also indicate that RodA1 is the “main” RodA enzyme in L. monocytogenes, as no significant phenotypic changes with regard to growth and cell division could be observed as long as RodA1 was present. On the other hand, RodA2 was able to compensate for the loss of RodA1 and RodA3 only when it was overproduced from an inducible promoter. Interestingly, cells of strains 10403S and 10403SΔ*rodA1*Δ*rodA3* in which *rodA3* is overexpressed from an ectopic locus have an increased cell length compared to the wild type. An explanation for this could be that elevated levels of RodA3 lead to the depletion of proteins needed at the cell division site, resulting in an extended synthesis of PG on the lateral wall. Another possibility could be that RodA3 directly inhibits FtsW1 or displaces FtsW1 at the cell division site, leading to a block in cell division and therefore resulting in the formation of elongated cells.

Recent studies have shown that SEDS proteins act as glycosyltransferases (22, 24). The glycosyltransferase activity of PBPs and MGT can be inhibited by moenomycin, whereas RodA/FtsW enzymes are not affected by moenomycin and are therefore important for moenomycin resistance (24, 61). In good agreement with the importance of SEDS proteins for the intrinsic moenomycin resistance, deletion of the genes encoding two of the three RodA enzymes, RodA1 and RodA3, resulted in an increased moenomycin sensitivity of L. monocytogenes (Fig. 6C).

In B. subtilis, RodA is in a complex with the class B PBP, PBP 2A (also named PbpH), and these two proteins act together to polymerize and cross-link the glycan strands (34, 56). Similarly, FtsW and PBP 2B form a subcomplex as part of the divisome (57, 58). Recently, it was shown that RodA-PBP3 and FtsW-PBP1 act as cognate pairs in the coccoid bacterium S. aureus (28). Depletion of all three RodA enzymes in L. monocytogenes, RodA1, RodA2, and RodA3, leads to a drastic reduction in cell length (Fig. 5). A similar phenotype was observed for an L. monocytogenes strain depleted for the essential class B PBP, PBP B1 (51). In contrast, the absence of either FtsW1 (Fig. 2) or the class B PBP, PBP B2, in L. monocytogenes results in the formation of elongated cells (51). These observations suggest that RodA and FtsW might work in a complex with the cognate PBPs PBP B1 and PBP B2 during cell elongation and cell division, respectively. Indeed, protein-protein interactions between FtsW1 and FtsW2 with PBP B2 and between RodA1, RodA2, and RodA3 with the PBP B1 could be observed (Fig. 8). However, interactions were also detected between the FtsW proteins and PBP B1 and PBP B3 as well as between the RodA proteins and PBP B2 and PBP B3 (Fig. 8). While these data provide the first line of evidence that SEDS proteins and class B PBPs (bPBP) also form complexes in L. monocytogenes, additional work is necessary to determine whether specific SEDS-bPBP pairs are formed in L. monocytogenes.

Taken together, L. monocytogenes has a repertoire of PBPs and multiple members of the SEDS family of proteins to produce its rigid cell wall. The expression and activity of these enzymes need to be tightly regulated in L. monocytogenes to maintain its cell shape. Our results suggest that L. monocytogenes adapts the expression of a second set of FtsW/RodA enzymes, FtsW2 and RodA3, to environmental stresses such as the presence of β-lactam antibiotics, thereby preventing defects in the peptidoglycan synthesis and subsequent cell lysis.

## MATERIALS AND METHODS

### Bacterial strains and growth conditions.

All strains and plasmids used in this study are listed in Table S1 in the supplemental material. Strain and plasmid constructions are described in Text S1 in the supplemental material, and all primers used in this study are listed in Table S2. E. coli strains were grown in Luria-Bertani (LB) medium, and L. monocytogenes strains were grown in brain heart infusion (BHI) medium at 37°C unless otherwise stated. If necessary, antibiotics and supplements were added to the medium at the following concentrations: for E. coli cultures, ampicillin (Amp) at 100 μg/ml and kanamycin (Kan) at 30 μg/ml, and for L. monocytogenes cultures, chloramphenicol (Cam) at 10 μg/ml, kanamycin (Kan) at 30 μg/ml, and isopropyl β-d-1-thiogalactopyranoside (IPTG) at 1 mM. We used the L. monocytogenes strain 10403S and derivatives thereof. However, we refer to L. monocytogenes EGD-e gene and locus tag numbers, as this was the first fully sequenced L. monocytogenes strain.

10.1128/mBio.01448-19.7 TEXT S1 Supplemental Materials and Methods. Download Text S1, DOCX file, 0.02 MB. Copyright © 2019 Rismondo et al. 2019 Rismondo et al. This content is distributed under the terms of the Creative Commons Attribution 4.0 International license.

10.1128/mBio.01448-19.8 TABLE S1 Bacterial strains used in this study. Download Table S1, DOCX file, 0.03 MB. Copyright © 2019 Rismondo et al. 2019 Rismondo et al. This content is distributed under the terms of the Creative Commons Attribution 4.0 International license.

10.1128/mBio.01448-19.9 TABLE S2 Primers used in this study. Download Table S2, DOCX file, 0.02 MB. Copyright © 2019 Rismondo et al. 2019 Rismondo et al. This content is distributed under the terms of the Creative Commons Attribution 4.0 International license.

### Growth curves.

Overnight cultures of wild-type L. monocytogenes 10403S and the indicated deletion strains were diluted to an optical density at 600 nm (OD_600_) of 0.01 or 0.05 in 15 ml of BHI medium, and the cultures were incubated at 37°C with shaking. Growth was monitored by determining OD_600_ readings at hourly intervals. For growth curves with the IPTG-inducible depletion strains 10403SΔ*ftsW1* i*ftsW* (ANG4314), 10403SΔ*ftsW1* i*ftsW2* (ANG5119), and 10403SΔ*rodA1-3* i*rodA1* (ANG5192), the strains were cultivated overnight in the presence of 1 mM IPTG. The next day, cells were washed once with fresh medium, diluted 1:50 in 5 ml BHI medium, and grown for 8 to 10 h in the absence of the inducer. The cultures were diluted 1:100 into fresh BHI medium and grown until the next morning at 37°C. The depleted cells were then diluted to an OD_600_ of 0.01 and grown in the presence or absence of 1 mM IPTG at 37°C. Averages and standard deviations from three independent experiments were plotted.

### RNA extraction and quantitative real-time PCR (qRT-PCR).

For the extraction of RNA from *rodA* complemented strains, overnight cultures of L. monocytogenes strains 10403S, 10403SΔ*rodA1*Δ*rodA3*, 10403SΔ*rodA1*Δ*rodA3* pIMK3-*rodA1*, 10403SΔ*rodA1*Δ*rodA3* pIMK3-*rodA2*, and 10403SΔ*rodA1*Δ*rodA3* pIMK3-*rodA3* were diluted in BHI medium (with 1 mM IPTG for the plasmid-containing complemented strains) to an OD_600_ of 0.05 and incubated at 37°C until the cultures reached an OD_600_ of 1. For the extraction of RNA from strain 10403SΔ*rodA1*-*3* i*rodA1* (ANG5192), bacteria were grown as described for the growth curve assay to deplete RodA1. Next, strain 10403S and depleted cells of 10403SΔ*rodA1*-*3* i*rodA1* were diluted to an OD_600_ of 0.01 and grown in BHI medium in the presence or absence of IPTG until an OD_600_ of 0.5. Portions (20 ml) of the cultures were mixed with 47 ml guanidine thiocyanate (GTC) buffer (5 M GTC, 0.5% *N*-lauryl sarcosine, 0.1 M β-mercaptoethanol, 0.5% Tween 80, 10 mM Tris-HCl [pH 7.5]), bacteria were harvested by centrifugation and subsequently lysed using the FastRNA Pro Blue kit (MP Biomedicals). Total RNA was isolated by chloroform extraction and ethanol precipitation and further purified using the RNeasy minikit (Qiagen) and finally treated with Turbo DNase (Invitrogen). cDNA was synthesized from 10 ng of RNA using the Superscript III first strand synthesis kit (Invitrogen). The expression of *rodA1*, *rodA2*, and *rodA3* in the different strains was assessed using the TaqMan probe-based gene expression assay (Applied Biosystems). Expression of *gyrB* was used as a control. The cycle threshold (*C_T_*) values obtained for *rodA1*, *rodA2* and *rodA3* were normalized to the values obtained for *gyrB*. The fold changes of gene expression for the different strains were calculated using the ΔΔ*C_T_* method.

### Determination of MICs.

The MICs for bacitracin, penicillin, moenomycin, and lysozyme were determined using a broth microdilution assay in 96-well plates. Approximately 10^4^ L. monocytogenes cells were used to inoculate 200 μl BHI containing twofold dilutions of the different antimicrobials. The starting antibiotic concentrations were 1 mg/ml for bacitracin A, 1 μg/ml for penicillin G, 0.8 or 1.6 μg/ml for moenomycin, 8 μg/ml cefuroxime, and 10 mg/ml for lysozyme. The OD_600_ readings were determined after incubating the 96-well plates for 24 h at 37°C with shaking at 500 rpm in a plate incubator (Thermostar; BMG Labtech). The MIC value refers to the antibiotic concentration at which bacterial growth was inhibited by >90%.

### Determination of antibiotic susceptibility using a spot plating assay.

Overnight cultures of the indicated L. monocytogenes strains were adjusted to an OD_600_ of 1, and 5-μl portions of serial dilutions were spotted on BHI agar plates or BHI agar plates containing 1 μg/ml cefuroxime and where indicated, 1 mM IPTG. Plates were photographed after incubation at 37°C for 24 h.

### Fluorescence and phase-contrast microscopy.

For bacterial cell length measurements, 100-μl portions of mid-log cultures were mixed with 5 μl of 100 μg/ml Nile red solution to stain the cell membrane. Following incubation at 37°C for 20 min, the cells were washed once with phosphate-buffered saline (PBS) and resuspended in 50 μl PBS. Portions (1.5 μl) of the different samples were spotted onto microscope slides covered with a thin agarose film (1.5% agarose in distilled water), air dried, and covered with a cover slip. Phase-contrast and fluorescence images were taken using a Zeiss Axio Imager.A1 microscope coupled to an AxioCam MRm and a 100× objective and processed using the Zen 2012 software (blue edition). For the detection of Nile red fluorescence signals, the Zeiss filter set 00 was used. For the cell length determinations, 300 cells were measured for each experiment, and the median cell length was calculated. Averages and standard deviations of the median cell length of three independent experiments were plotted.

### Peptidoglycan isolation and analysis.

Overnight cultures of L. monocytogenes strains 10403S, 10403SΔ*rodA1*Δ*rodA3*, and 10403SΔ*rodA1*Δ*rodA3* pIMK3-*rodA1* were used to inoculate 1 liter BHI broth (with 1 mM IPTG for the complementation strain 10403SΔ*rodA1*Δ*rodA3* pIMK3-*rodA1*) to a starting OD_600_ of 0.06. The cultures were grown at 37°C until they reached an OD_600_ of 1, at which point the cultures were cooled on ice for 1 h. The bacteria were subsequently collected by centrifugation, and peptidoglycan was purified and digested with mutanolysin as described previously (62, 63). Digested muropeptides were analyzed by high-performance liquid chromatography (HPLC) and recorded at an absorption of 205 nm as described previously (62). For quantification, the areas of the main muropeptide peaks were integrated using the Agilent Technology ChemStation software. The sum of the peak areas was set at 100%, and individual peak areas were determined. Averages and standard deviations from three independent extractions were calculated.

### β-Galactosidase assay.

For determination of the β-galactosidase activity, overnight cultures of strains 10403S pPL3e-P*_lmo2689_-lacZ*, 10403SΔ*rodA1* pPL3e-P*_lmo2689_-lacZ*, and 10403SΔ*rodA1*Δ*rodA2* pPL3e-P*_lmo2689_-lacZ* were diluted 1:100 in fresh BHI medium and grown for 6 h at 37°C. Sample collection and preparation were performed as described previously (64). Briefly, OD_600_ readings were determined (for the final β-galactosidase unit calculations) for the different cultures after 6 h of growth, and cells from 1 ml culture were pelleted by centrifugation for 10 min at 13,200 × *g*, resuspended in 100 μl ABT buffer (60 mM K_2_HPO_4_, 40 mM KH_2_PO_4_, 100 mM NaCl, 0.1% Triton X-100 [pH 7.0]), snap-frozen in liquid nitrogen, and stored at −80°C until use. For the identification of substances inducing the expression of the *lmo2689-lmo2686* operon, an overnight culture of strain 10403S pPL3e-P*_lmo2689_-lacZ* was diluted 1:100 in fresh BHI medium, and the culture was incubated with shaking at 37°C until an OD_600_ of 0.5 to 0.6 was reached. The culture was divided into several flasks and incubated for 2 h at 37°C in the presence or absence of the following substances: 0.5 μg/ml ampicillin, 0.05 μg/ml penicillin, 0.5 μg/ml vancomycin, 4 μg/ml cefuroxime, 0.05 μg/ml moenomycin, 0.5 mg/ml lysozyme, 1% ethanol, 300 μg/ml MgSO_4_, or 300 μg/ml EDTA. Bacteria were pelleted, and samples were frozen as described above.

Samples were thawed, and 1:10 dilutions were prepared in ABT buffer. Fifty-microliter portions of the 1:10 diluted samples were mixed with 10 μl of 0.4 mg/ml 4-methyl-umbelliferyl-β-d-galactopyranoside (MUG) substrate prepared in dimethyl sulfoxide (DMSO) and incubated for 60 min at room temperature (RT). A reaction with ABT buffer alone was used as a negative control. Following this incubation step, 20 μl of each reaction mixture was diluted into 180 μl of ABT buffer in a black 96-well plate, and fluorescence values were measured using an Hidex Sense microplate reader at 355-nm excitation and 460-nm emission wavelengths. Concentrations from 0.125 μM to 20 μM of the fluorescent 4-methylumbelliferone (MU) standard were used to obtain a standard curve. β-Galactosidase units were calculated as (picomoles of substrate hydrolyzed × dilution factor)/(culture volume [in milliliters] × OD_600_ × minute). The amount of hydrolyzed substrate was determined from the standard curve as (emission reading – *y* intercept)/slope.

### Bacterial two-hybrid assays.

Protein-protein interactions between the different FtsW/RodA homologs and class B PBPs were analyzed using the bacterial adenylate cyclase two-hybrid (BACTH) assay (65). The indicated pUT18/pUT18c and pKT25 derivatives were cotransformed into E. coli strain BTH101. Transformants were selected on LB agar plates containing 100 μg/ml ampicillin, 30 μg/ml kanamycin, 0.1 mM IPTG, and 50 μg/ml X-Gal. Images were taken after incubation for 24 h and 48 h at 30°C.
