# Supplementary figures and images for: Cell Shape and Antibiotic Resistance Are Maintained by the Activity of Multiple FtsW and RodA Enzymes in Listeria monocytogenes
Source: mBio. 2019 Aug 6;10(4):e01448-19. doi: 10.1128/mBio.01448-19 (PMC6686043; doi:10.1128/mBio.01448-19)

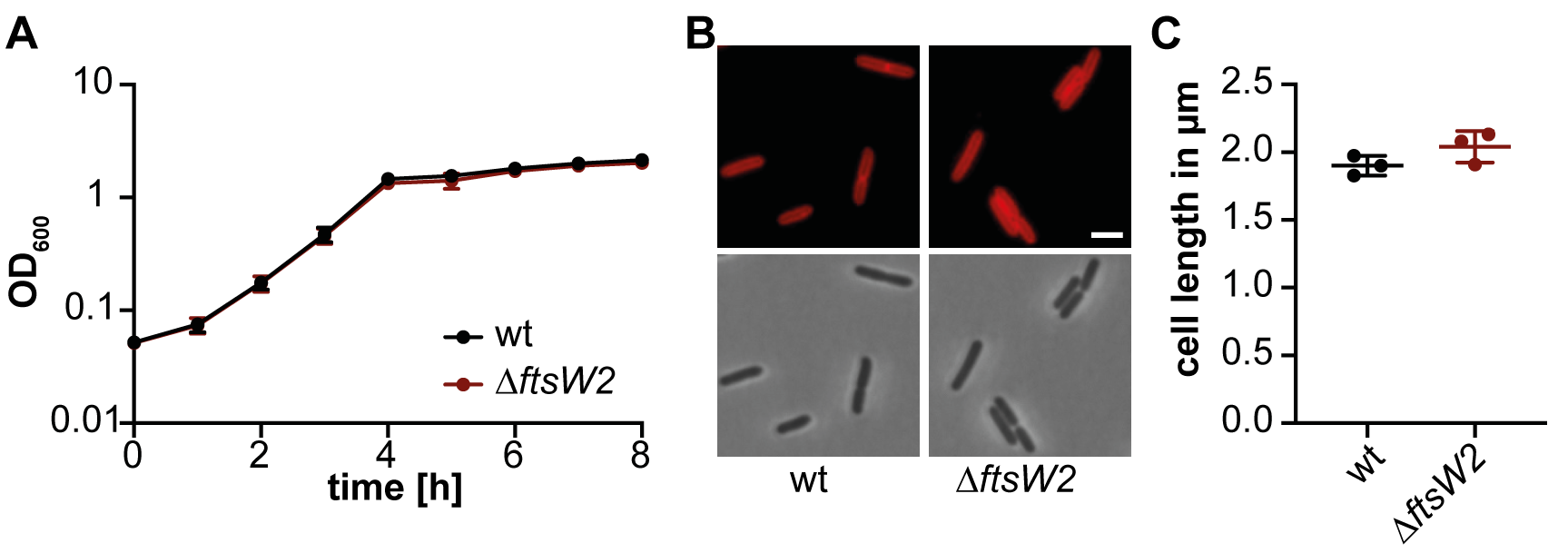

Supplement: FIG S2 [file mBio.01448-19-sf002.tif]

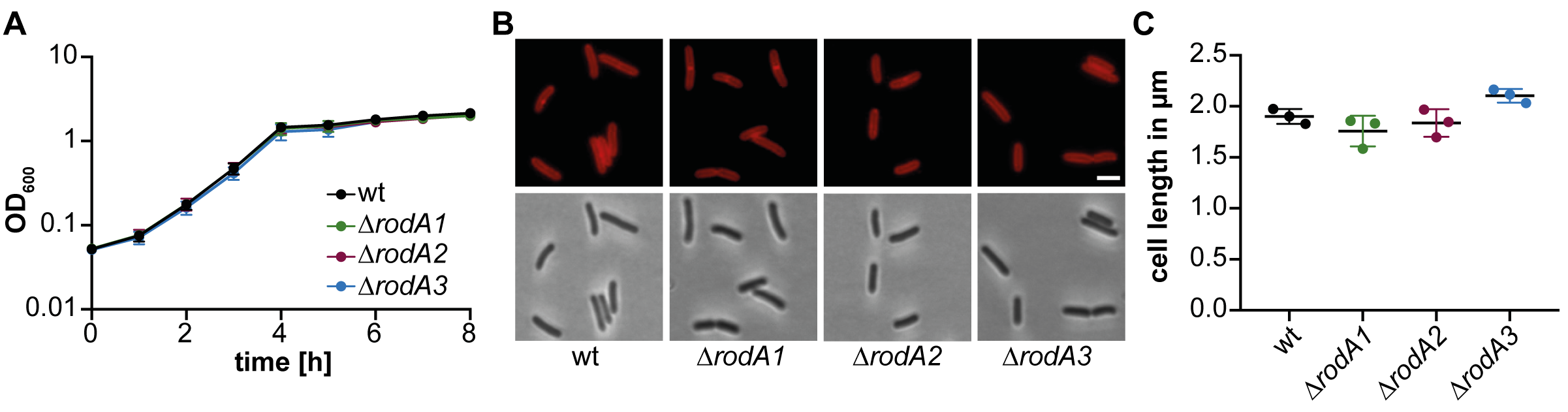

Supplement: FIG S3 [file mBio.01448-19-sf003.tif]

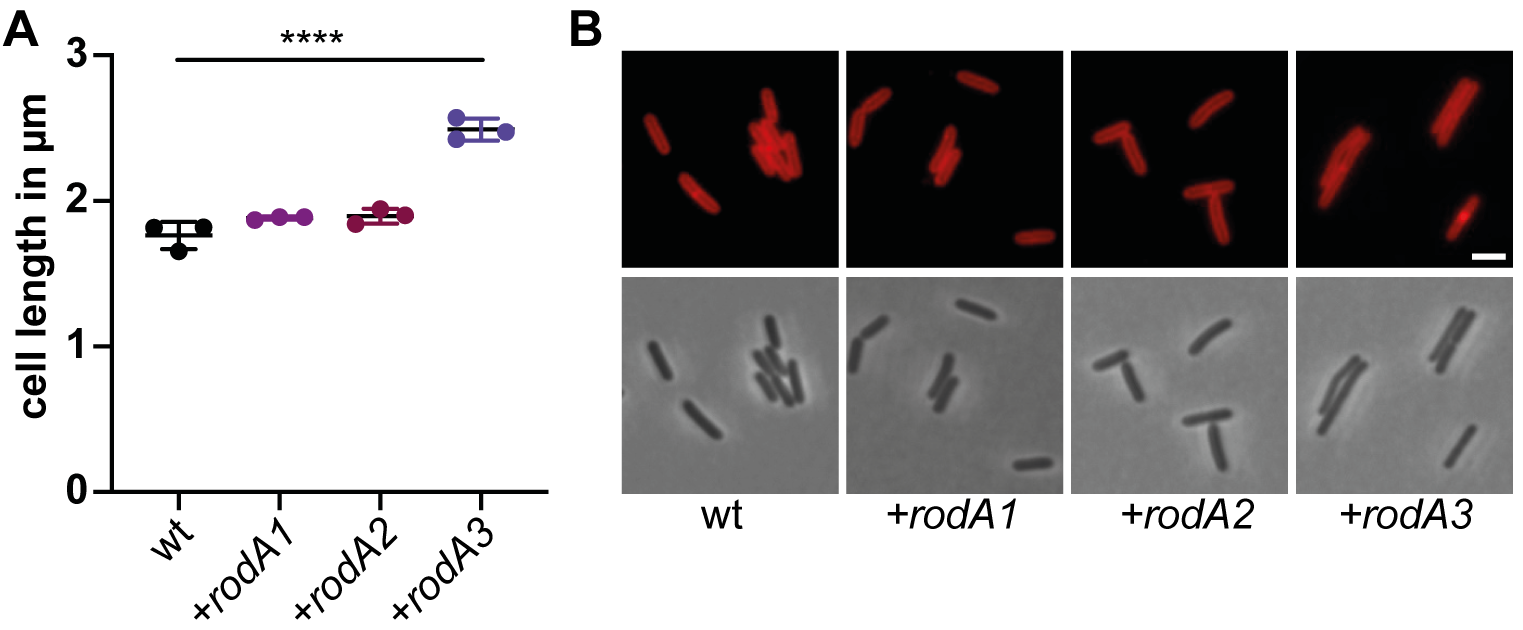

Supplement: FIG S4 [file mBio.01448-19-sf004.tif]

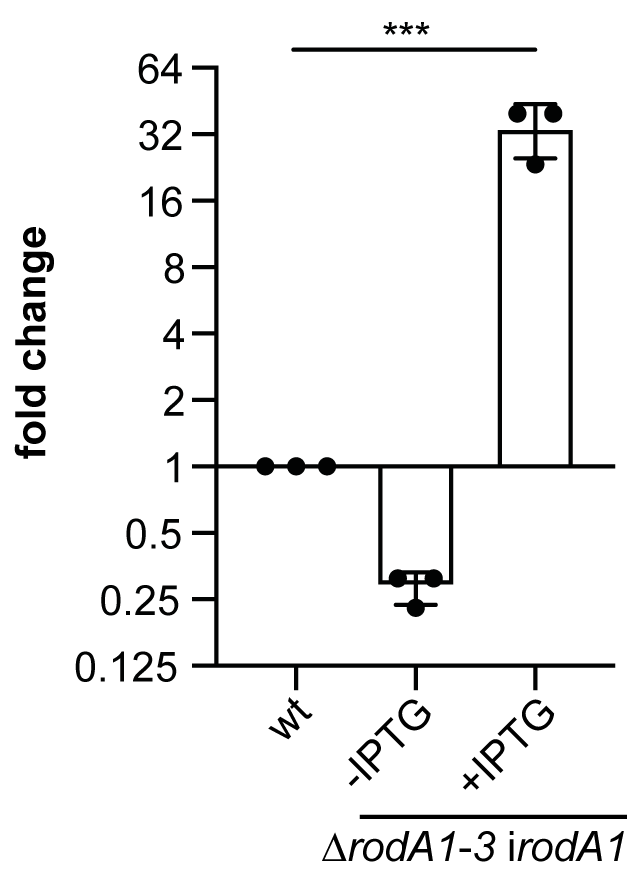

Supplement: FIG S5 [file mBio.01448-19-sf005.tif]

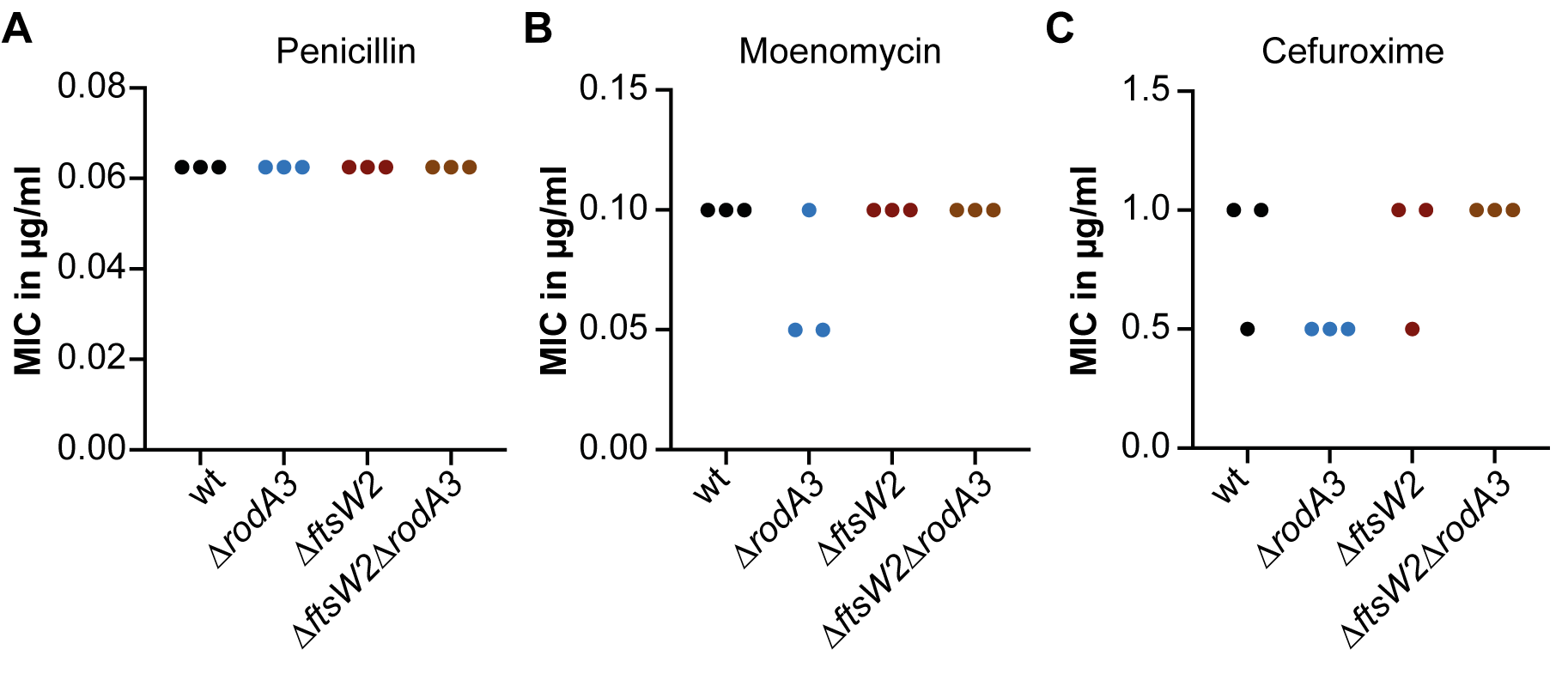

Supplement: FIG S6 [file mBio.01448-19-sf006.tif]
