# Supplementary material for: Cell Shape and Antibiotic Resistance Are Maintained by the Activity of Multiple FtsW and RodA Enzymes in Listeria monocytogenes
Source: mBio. 2019 Aug 6;10(4):e01448-19. doi: 10.1128/mBio.01448-19 (PMC6686043; doi:10.1128/mBio.01448-19)
Supplement: TABLE S1 [file mBio.01448-19-st001.docx]

Supplementary information for:

**Cell shape and antibiotic resistance is maintained by the activity of multiple FtsW and RodA enzymes in *Listeria monocytogenes***

**Jeanine Rismondo^1^, Sven Halbedel^2^ and Angelika Gründling^1^**

^1^ Section of Microbiology and Medical Research Council Centre for Molecular Bacteriology and Infection, Imperial College London, London SW7 2AZ.

^2^ FG11 Division of Enteropathogenic bacteria and *Legionella*, Robert Koch-Institute, Burgstrasse 37, 38855 Wernigerode, Germany.

**Table S1: Bacterial strains used in this study**

| **Unique ID** | **Strain name and resistance** | **Source** |
| --- | --- | --- |
| ***Escherichia coli* strains** | |  |
| ANG1264 | DH5α pKSV7; AmpR | (1) |
| ANG1278 | XL1-Blue pPL3e; CamR | (2) |
| ANG2027 | XL1-Blue p*itet*-P_700-_*_ltaS_*-*lacZ*, AmpR | Lab strain collection |
| ANG4243 | XL1-Blue pIMK3; KanR | (3) |
| ANG4132 | XL1-Blue pKSV7-∆*rodA2*; AmpR | This study |
| ANG4135 | XL1-Blue pKSV7-∆*rodA1*; AmpR | This study |
| ANG4137 | XL1-Blue pKSV7-∆*ftsW2*; AmpR | This study |
| ANG4169 | CLG190 pKSV7-∆*ftsW1*; AmpR | This study |
| ANG4261 | XL1-Blue pIMK3-*ftsW1*; KanR | This study |
| ANG4444 | XL1-Blue pKSV7-∆*rodA1*∆*rodA2*; AmpR | This study |
| ANG4457 | XL1-Blue pKSV7-∆*ftsW2*∆*rodA3*; AmpR | This study |
| ANG4665 | XL1-Blue pKSV7-∆*rodA3*; AmpR | This study |
| ANG4892 | XL1-Blue pIMK3-*rodA1*; KanR | This study |
| ANG4893 | XL1-Blue pIMK3-*rodA3*; KanR | This study |
| ANG4976 | XL1-Blue pIMK3-*rodA2*; KanR | This study |
| ANG4977 | XL1-Blue pIMK3-*ftsW2*; KanR | This study |
| ANG5181 | XL1-Blue pPL3e-*lacZ*; CamR | This study |
| ANG5193 | XL1-Blue pPL3e-P_600-_*_lmo2689_-lacZ*; CamR | This study |
| ANG5402 | XL1 Blue pKT25; KanR | (4) |
| ANG5415 | XL1 Blue pUT18; AmpR | (4) |
| ANG5416 | XL1 Blue pUT18c; AmpR | (4) |
| pJR201 | TOP10 pUT18-*rodA1*; AmpR | This study |
| pJR202 | TOP10 pUT18-*rodA3*; AmpR | This study |
| pJR204 | TOP10 pUT18-*rodA2*; AmpR | This study |
| pJR205 | TOP10 pUT18-*ftsW2*; AmpR | This study |
| pJR206 | TOP10 pUT18-*ftsW1*; AmpR | This study |
| pJR207 | TOP10 pUT18c-*rodA1*; AmpR | This study |
| pJR208 | TOP10 pUT18c-*rodA3*; AmpR | This study |
| pJR210 | TOP10 pUT18c-*rodA2*; AmpR | This study |
| pJR211 | TOP10 pUT18c-*ftsW2*; AmpR | This study |
| pJR212 | TOP10 pUT18c-*ftsW1*; AmpR | This study |
| pSH235 | TOP10 pKT25-*pbpB2*; KanR | (5) |
| pSH236 | TOP10 pKT25-*pbpB1*; KanR | (5) |
| pSH237 | TOP10 pKT25-*pbpB3*; KanR | (5) |
| ***Listeria monocytogenes* strains** | |  |
| ANG1263 | 10403S; StrepR | (6) |
| ANG4171 | 10403S∆*rodA1*; StrepR | This study |
| ANG4172 | 10403S∆*rodA2*; StrepR | This study |
| ANG4176 | 10403S∆*ftsW2*; StrepR | This study |
| ANG4288 | 10403S pIMK3-*ftsW1*; StrepR KanR | This study |
| ANG4314 | 10403S∆*ftsW1* pIMK3-*ftsW1*; StrepR KanR, IPTG | This study |
| ANG4459 | 10403S∆*rodA1*∆*rodA2*; StrepR | This study |
| ANG4482 | 10403S∆*ftsW2*∆*rodA3*; StrepR | This study |
| ANG4683 | 10403S∆*rodA3*; StrepR | This study |
| ANG4684 | 10403S∆*rodA1*∆*rodA3*; StrepR | This study |
| ANG4685 | 10403S∆*rodA2*∆*rodA3*; StrepR | This study |
| ANG4905 | 10403S∆*rodA1*∆*rodA3* pIMK3-*rodA1*; StrepR KanR | This study |
| ANG4906 | 10403S∆*rodA1*∆*rodA3* pIMK3-*rodA3*; StrepR KanR | This study |
| ANG4966 | 10403S pIMK3-*rodA1*; StrepR KanR | This study |
| ANG4967 | 10403S pIMK3-*rodA3*; StrepR KanR | This study |
| ANG4981 | 10403S pIMK3-*rodA2*; StrepR KanR | This study |
| ANG4982 | 10403S pIMK3-*ftsW2*; StrepR KanR | This study |
| ANG5119 | 10403S∆*ftsW1* pIMK3-*ftsW2*; StrepR KanR, IPTG | This study |
| ANG5148 | 10403S∆*rodA1*∆*rodA2* pIMK3-*rodA1*; StrepR KanR | This study |
| ANG5149 | 10403S∆*rodA1*∆*rodA3* pIMK3-*rodA2*; StrepR KanR | This study |
| ANG5192 | 10403S∆*rodA1-3* pIMK3-*rodA1*; StrepR KanR, IPTG | This study |
| ANG5198 | 10403S pPL3e-P*_lmo2689_*-*lacZ*; StrepR ErmR | This study |
| ANG5292 | 10403S∆*rodA1* pPL3e-P*_lmo2689_*-*lacZ*; StrepR ErmR | This study |
| ANG5293 | 10403S∆*rodA1*∆*rodA2* pPL3e-P*_lmo2689_*-*lacZ*; StrepR ErmR | This study |

1. Smith K, Youngman P. 1992. Use of a new integrational vector to investigate compartment-specific expression of the *Bacillus subtilis spoIIM* gene. Biochimie 74:705-11.

2. Gründling A, Burrack LS, Bouwer HG, Higgins DE. 2004. *Listeria monocytogenes* regulates flagellar motility gene expression through MogR, a transcriptional repressor required for virulence. Proc Natl Acad Sci U S A 101:12318-23.

3. Monk IR, Gahan CG, Hill C. 2008. Tools for functional postgenomic analysis of *Listeria monocytogenes*. Appl Environ Microbiol 74:3921-34.

4. Karimova G, Pidoux J, Ullmann A, Ladant D. 1998. A bacterial two-hybrid system based on a reconstituted signal transduction pathway. Proc Natl Acad Sci U S A 95:5752-6.

5. Cleverley RM, Rutter ZJ, Rismondo J, Corona F, Tsui HT, Alatawi FA, Daniel RA, Halbedel S, Massidda O, Winkler ME, Lewis RJ. 2019. The cell cycle regulator GpsB functions as cytosolic adaptor for multiple cell wall enzymes. Nat Commun 10:261.

6. Bishop DK, Hinrichs DJ. 1987. Adoptive transfer of immunity to *Listeria monocytogenes*. The influence of in vitro stimulation on lymphocyte subset requirements. J Immunol 139:2005-9.
